# Supplementary material for: Polyunsaturated Aldehydes Profile in the Diatom Cyclotella cryptica Is Sensitive to Changes in Its Phycosphere Bacterial Assemblages
Source: Mar Drugs. 2023 Oct 30;21(11):571. doi: 10.3390/md21110571 (PMC10672285; doi:10.3390/md21110571)
Supplement: Supplementary file 1 [file marinedrugs-21-00571-s001.zip › marinedrugs-2637012-supplementary.pdf]

Supplementary Material

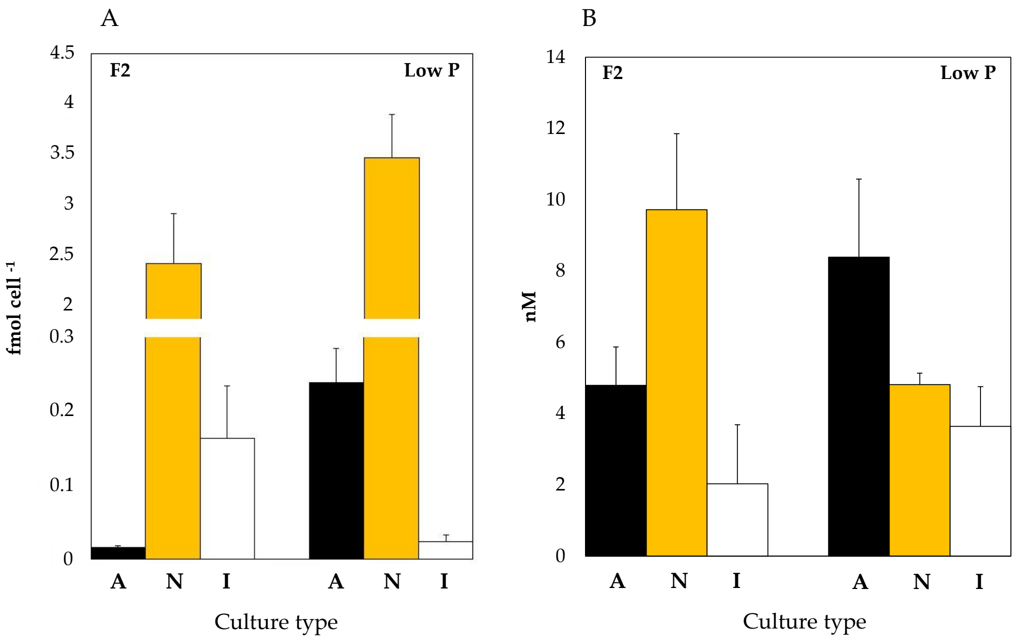

Figure S1. Total pPUA (A) and total dPUA (B) concentrations at the *C. cryptica* cultures in late exponential growth phase at the two phosphate availability conditions assayed (F2 and low P). pPUA scale is normalized by cell density. Error bars are the standard deviation (n=5). Sample key: A-cultures = Axenic cultures of *C. cryptica*; N-cultures = *C. cryptica* co-cultured with autoctonous heterotrophic bacteria; I-cultures = *C. cryptica* co-cultured with introduced heterotrophic bacteria.

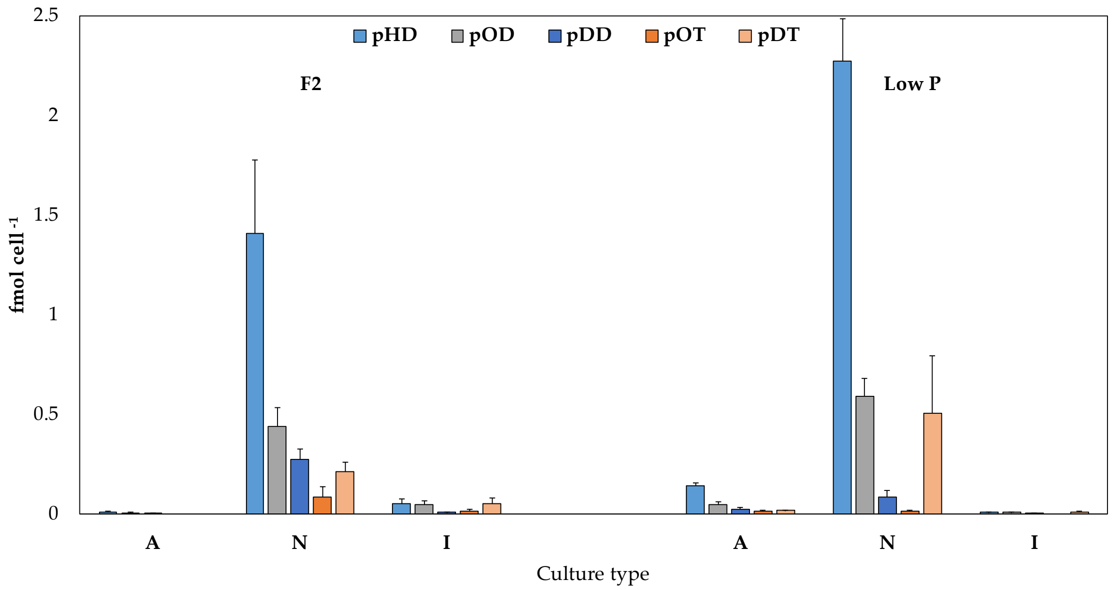

Figure S2. Average pPUA types concentration detected in *C. cryptica* cultures quantified at late exponential growth phase at the two phosphate availability conditions assayed (F2 and low P). Sample key: A-cultures = Axenic cultures of *C. cryptica*; N-cultures = *C. cryptica* co-cultured with autoctonous heterotrophic bacteria; I-cultures = *C. cryptica* co-cultured with introduced heterotrophic bacteria. pHD = particulate *2E,4E/Z*-heptadienal; pOD = particulate *2E,4E/Z*-octadienal; pDD = particulate *2E,4E/Z*-decadienal; pOT = particulate *2E,4E/Z,7*-octatrienal; pDT = particulate *2E,4E/Z,7Z*-decatrienal. pPUA data are normalized by cell density (n=5).

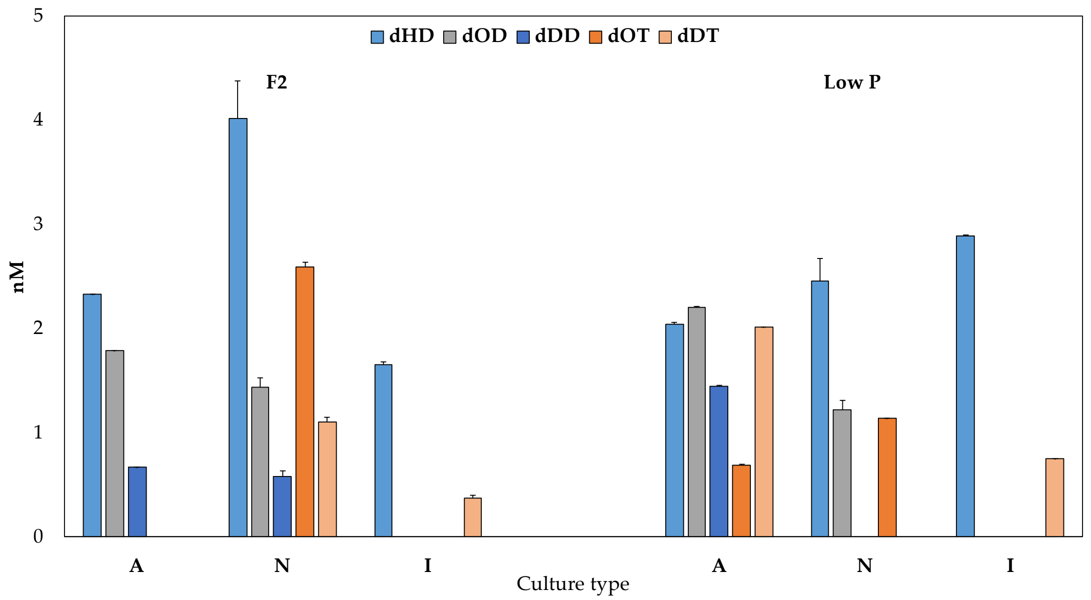

Figure S3. Average dPUA types concentration detected in *C. cryptica* cultures cultures quantified in late exponential growth phase at the two phosphate availability conditions assayed (F2 and low P) (n=5). Sample key: A-cultures = Axenic cultures of *C. cryptica*; N-cultures = *C. cryptica* co-cultured with autoctonous heterotrophic bacteria; I-cultures = *C. cryptica* co-cultured with introduced heterotrophic bacteria.; dHD = dissolved 2*E*,4*E*/*Z*-heptadienal; dOD = dissolved 2*E*,4*E*/*Z*-octadienal; dDD = dissolved 2*E*,4*E*/*Z*-decadienal; dOT = dissolved 2*E*,4*E*/*Z*,7-octatrienal; dDT = dissolved 2*E*,4*E*/*Z*,7*Z*-decatrienal (n=5).

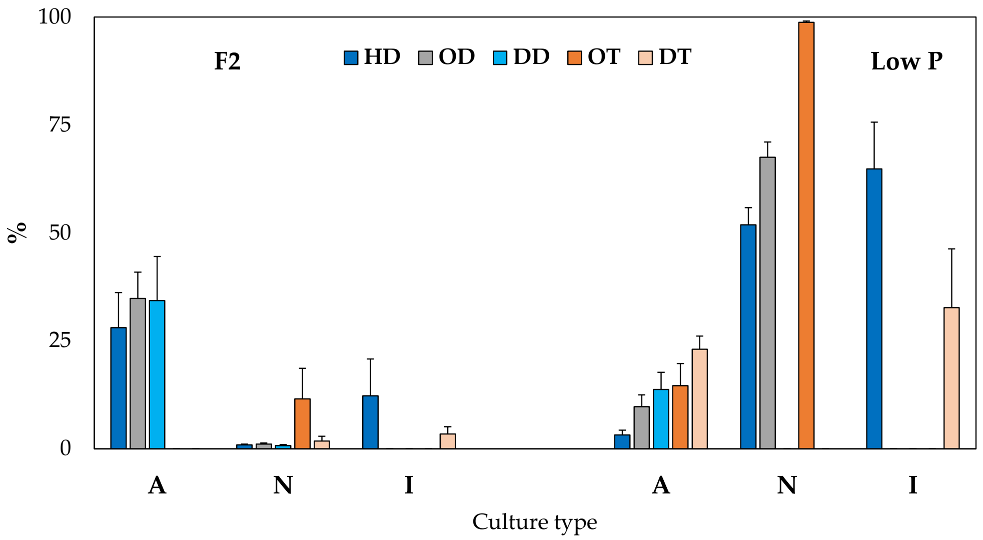

Figure S4. Dissolved PUA types percentage in the different *C. cryptica* cultures quantified in late exponential growth phase at the two phosphate availability conditions assayed (F2 and low P). The percentage was obtained adding the concentrations of total pPUA types and total dPUA types (nM). Sample key: A-cultures = Axenic cultures of *C. cryptica*; N-cultures = *C. cryptica* co-cultured with autoctonous heterotrophic bacteria; I-cultures = *C. cryptica* co-cultured with introduced heterotrophic bacteria. HD = 2*E*,4*E*/*Z*-heptadienal; OD = 2*E*,4*E*/*Z*-octadienal; DD = 2*E*,4*E*/*Z*-decadienal; OT = 2*E*,4*E*/*Z*,7-octatrienal; DT = 2*E*,4*E*/*Z*,7*Z*-decatrienal (n=5).

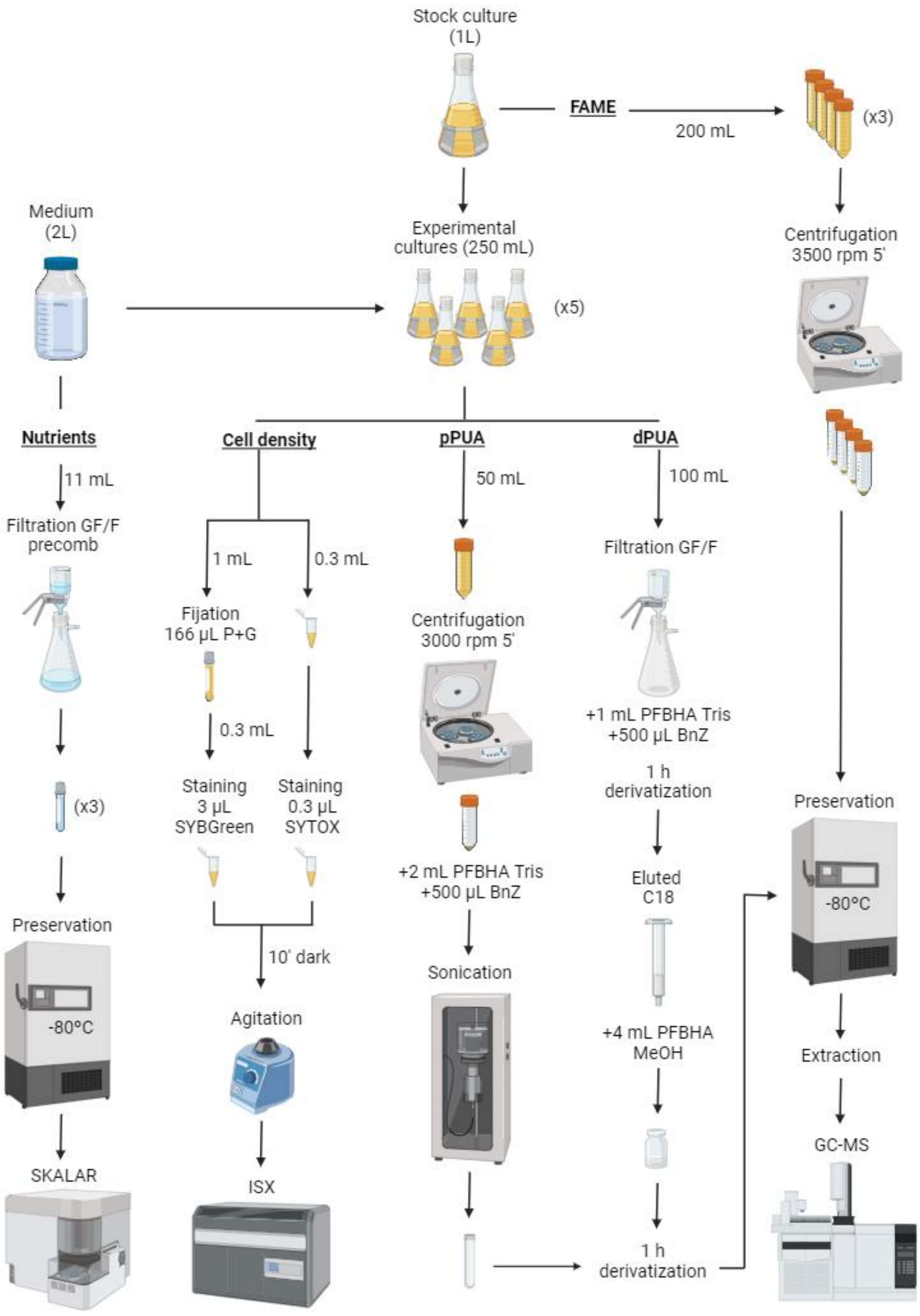

Figure S5. Flow diagram of the experimental procedures carried out in this study. The steps carried out in the experimental sampling and the subsequent procedures to obtain the variables of interest (nutrient, cell density, pPUA, dPUA and FAME concentrations) are shown. Sample key: P+G= paraformaldehyde and glutaraldehyde; PFBHA= reagent *O*-(2,3,4,5,6-pentafluorobenzylhydroxylamine)hydrochloride; Tris= Tris-HCl; BnZ= benzaldehyde, internal standard; C18= LiChrolut® RP C-18 cartridge; MeOH= methanol; SKALAR= Skalar autoanalyzer; ISX= Luminex ImageStream ® X Mk II; GC-MS= gas chromatography-mass spectrometer.

Table S1: Results of one-way ANOVA tests between growth rates of *C. cryptica* and heterotrophic bacteria present in the cultures at the two phosphate availability conditions assayed (F2 and low P) (n=5). Sample key: A-cultures = Axenic cultures of *C. cryptica*; N-cultures = *C. cryptica* co-cultured with autoctonous heterotrophic bacteria; I-cultures = *C. cryptica* co-cultured with introduced heterotrophic bacteria.

**Growth rates (day^{-1})**					
**One-way ANOVA**					
***C. cryptica* cultures**	**Conditions**	**Difference**	**F value**	**p-value**	**significance**
F2	A-N	-0.298	81.82	<0.001	***
	A-I	-0.497	21.24	0.002	**
Low P	A-N	-0.105	6.24	0.041	*
	A-I	-0.302	214.92	<0.001	***
**Heterotrophic bacteria**	**Conditions**	**Difference**	**F value**	**p-value**	**significance**
F2	N-I	-1.903	804.70	<0.001	***
Low P	N-I	-1.444	397.05	<0.001	***

Table S2: Results of one-way ANOVA tests between total pPUA concentrations of *C. cryptica* cultures quantified in late exponential growth phase at the two phosphate availability conditions assayed (F2 and low P) (n=5). Sample key: A-cultures = Axenic cultures of *C. cryptica*; N-cultures = *C. cryptica* co-cultured with autoctonous heterotrophic bacteria; I-cultures = *C. cryptica* co-cultured with introduced heterotrophic bacteria.

**Total pPUA (fmol cell^{-1})**					
**One-way ANOVA**					
	**Conditions**	**Difference**	**F value**	**p-value**	**significance**
F2	A-N	-2.40	74.08	<0.001	***
	A-I	-0.14	16.72	0.004	**
Low P	A-N	-3.22	219.6	<0.001	***
	A-I	0.22	103.7	<0.001	***

Table S3. Two-way ANOVA **s**tatistical analysis of the total pPUA (fmol cell^{-1}) concentration of *C. cryptica* cultures quantified in late exponential growth phase at the two phosphate availability conditions assayed (F2 and low P) (n=5). Sample key: A-cultures = Axenic cultures of *C. cryptica*; N-cultures = *C. cryptica* co-cultured with autoctonous heterotrophic bacteria; I-cultures = *C. cryptica* co-cultured with introduced heterotrophic bacteria; DF = Degree freedom.

**Total pPUA (fmol cell $^{-1}$)**					
**Two-way ANOVA**	**Factor**	**DF**	**F value**	**p-value**	**significance**
Total pPUA	bacterial presence	2	325.939	< 0.001	***
	phosphate availability	1	13.339	< 0.01	**
	interaction	2	11.639	< 0.001	***
**Post hoc**	**Conditions**	**Difference**		**p-value**	**significance**
F2	A-N	-2.400		< 0.001	***
	A-I	-0.147		0.968	
	N-I	2.253		< 0.001	***
Low P	A-N	-3.226		< 0.001	***
	A-I	0.214		0.861	
	N-I	3.441		< 0.001	***

A two-way ANOVA was conducted to test the effects of phosphate availability and the presence or absence of bacteria. A post hoc (Tukey Test) was conducted when significant differences were

observed from the two-way ANOVA. Level of significance: * p-value < 0.05, ** p-value < 0.01, and *** p-value < 0.001.

Table S4: Results of one-way ANOVA tests between pPUA types concentrations of *C. cryptica* cultures quantified in late exponential growth phase at the two phosphate availability conditions assayed (F2 and low P) (n=5). Sample key: A-cultures = Axenic cultures of *C. cryptica*; N-cultures = *C. cryptica* co-cultured with autoctonous heterotrophic bacteria; I-cultures = *C. cryptica* co-cultured with introduced heterotrophic bacteria. pHD = particulate *2E,4E/Z*-heptadienal; pOD = particulate *2E,4E/Z*-octadienal; pDD = particulate *2E,4E/Z*-decadienal; pOT = particulate *2E,4E/Z,7*-octatrienal; pDT = particulate *2E,4E/Z,7Z*-decatrienal.

pPUA types (fmol cell^{-1})					
**One way ANOVA**					
**pHD**	**Conditions**	**Difference**	**F value**	**p-value**	**significance**
F2	A-N	-1.40	57.04	<0.001	***
	A-I	-0.04	12.08	0.010	*
Low P	A-N	-2.13	380.4	<0.001	***
	A-I	0.13	181.9	<0.001	***
**pOD**	**Conditions**	**Difference**	**F value**	**p-value**	**significance**
F2	A-N	-0.435	80.42	<0.001	***
	A-I	-0.035	14.41	0.006	**
Low P	A-N	-0.54	132.5	<0.001	***
	A-I	0.04	62.01	<0.001	***
**pDD**	**Conditions**	**Difference**	**F value**	**p-value**	**significance**
F2	A-N	-0.268	98.8	<0.001	***
	A-I	-0.004	12.88	0.008	**
Low P	A-N	0.006	10.26	0.015	*
	A-I	0.018	39.84	<0.001	***
**pOT**	**Conditions**	**Difference**	**F value**	**p-value**	**significance**
F2	A-N	-0.08	10.45	0.014	*
	A-I	-0.001	13.79	0.007	**
Low P	A-N	0	2.152	0.186	
	A-I	0.01	22.72	0.002	**
**pDT**	**Conditions**	**Difference**	**F value**	**p-value**	**significance**
F2	A-N	-0.21	87	<0.001	***
	A-I	-0.05	11.25	0.012	*
Low P	A-N	0.01	11.3	0.01	**
	A-I	0.005	5.073	0.059	*

Table S5: Results of one-way ANOVA tests between total dPUA concentrations of *C. cryptica* cultures quantified in late exponential growth phase at the two phosphate availability conditions assayed (F2 and low P) (n=5). Sample key: A-cultures = Axenic cultures of *C. cryptica*; N-cultures = *C. cryptica* co-cultured with autoctonous heterotrophic bacteria; I-cultures = *C. cryptica* co-cultured with introduced heterotrophic bacteria.

**Total dPUA (nM)**					
**One-way ANOVA**					
	**Conditions**	**Difference**	**F value**	**p-value**	**significance**
F2	A-N	-4.92	21.23	0.002	**
	A-I	2.76	9.779	0.014	*
Low P	A-N	3.58	13.06	0.006	**
	A-I	4.75	18.66	0.002	**

Table S6. Two-way ANOVA Statistical analysis of the total dPUA concentration of *C. cryptica* cultures quantified in late exponential growth phase at the two phosphate availability conditions assayed (F2 and low P) (n=5). Sample key: A-cultures = Axenic cultures of *C. cryptica*; N-cultures = *C. cryptica* co-cultured with autoctonous heterotrophic bacteria; I-cultures = *C. cryptica* co-cultured with introduced heterotrophic bacteria. DF = Degree freedom.

**Total dPUA (nM)**					
**Two-way ANOVA**	**Factor**	**DF**	**F value**	**p-value**	**significance**
	bacterial presence	2	23.410	< 0.001	***
Total dPUA	phosphate availability	1	0.0312	0.8614	
	interaction	2	20.323	< 0.001	***
**Post hoc**	**Conditions**	**Difference**		**p-value**	**significance**
	A-N	-4.928		<0.001	*
F2	A-I	2.691		0.092	
	N-I	7.691		< 0.001	***
	A-N	3.579		0.015	*
Low P	A-I	4.751		<0.001	***
	N-I	1.172		0.838	

A two-way ANOVA was conducted to test the effects of phosphate availability and the presence or absence of bacteria. A post hoc (Tukey Test) was conducted when significant differences were observed from the two-way ANOVA. Level of significance: * p-value < 0.05, ** p-value < 0.01, and *** p-value < 0.001.

Table S7: Results of one-way ANOVA tests between dPUA types concentrations of *C. cryptica* cultures quantified in late exponential growth phase at the two phosphate availability conditions assayed (F2 and low P) (n=5). Sample key: A-cultures = Axenic cultures of *C. cryptica*; N-cultures = *C. cryptica* co-cultured with autoctonous heterotrophic bacteria; I-cultures = *C. cryptica* co-cultured with introduced heterotrophic bacteria. dHD = dissolved *2E,4E/Z*-heptadienal; dOD = dissolved *2E,4E/Z*-octadienal; dDD = dissolved *2E,4E/Z*-decadienal; dOT = dissolved *2E,4E/Z,7*-octatrienal; dDT = dissolved *2E,4E/Z,7Z*-decatrienal; NaN = not a number.

**dPUA types (nM)**					
**One-way ANOVA**					
**dHD**	**Conditions**	**Difference**	**F value**	**p-value**	**significance**
F2	A-N	-1.68	7.897	0.023	*
	A-I	0.68	0.928	0.364	
Low P	A-N	-0.42	2.187	0.177	
	A-I	-0.85	2.579	0.147	
**dOD**	**Conditions**	**Difference**	**F value**	**p-value**	**significance**
F2	A-N	0.36	9.137	0.016	*
	A-I	1.79	251.6	<0.001	***
Low P	A-N	0.98	9.117	0.016	*
	A-I	2.20	45.66	<0.001	***
**dDD**	**Conditions**	**Difference**	**F value**	**p-value**	**significance**
F2	A-N	0.09	0.679	0.434	
	A-I	0.67	53.59	<0.001	***
Low P	A-N	1.45	87.3	<0.001	***
	A-I	1.45	87.3	<0.001	***
**dOT**	**Conditions**	**Difference**	**F value**	**p-value**	**significance**
F2	A-N	-2.59	229.7	<0.001	***
	A-I		NaN	NaN	
Low P	A-N	-0.45	60.09	<0.001	***
	A-I	0.69	141.5	<0.001	***
**dDT**	**Conditions**	**Difference**	**F value**	**p-value**	**significance**
F2	A-N	-1.10	26.48	<0.001	***
	A-I	-0.37	9.908	0.013	*
Low P	A-N	2.01	61.91	<0.001	***
	A-I	1.26	22.64	0.0014	**

Table S8. FAMEs concentrations detected in *C. cryptica* stock cultures at the two phosphate availability conditions assayed (F2 and low P). Averages and standard deviation (SD) of saturated fatty acid (SFA), monounsaturated fatty acid (MUFA) and polyunsaturated fatty acids (PUFA) are shown (n=3). In parenthesis % TFA.

		**Exponential growth phase**		**Stationary growth phase**	
	**SFA**	average (fmol cell^{-1})	sd	average (fmol cell^{-1})	sd
**F2**	C12:0 (Lauric)	0.04	0.03	0.28	0.11
	C13:0 (Tridecanoic)	0.01	0.00	0.01	0.00
	C14:0 (Myristic)	5.11	1.15	6.66	0.59
	C15:0 (Pentadecanoic)	1.13	0.50	1.47	0.10
	C16:0 (Palmitic)	27.17	10.46	23.94	1.60
	C18:0 (Stearic)	2.12	0.47	1.60	0.28
	C20:0 (Arachidic)	0.23	0.16	0.15	0.02
	C22:0 (Behenic)	0.17	0.05	0.08	0.01
	Total SAT	35.98 (30.82%)	12.30	34.19 (22.16%)	2.52
**Low P**	C14:0 (Myristic)	7.59	0.26	1.59	0.02
	C16:0 (Palmitic)	59.30	0.84	11.52	0.01
	C18:0 (Stearic)	17.05	0.04	0.52	0.01
	C20:0 (Arachidic)	0.22	0.01		
	Total SAT	84.16 (38.93%)	1.13	13.62 (32.75%)	0.02
	**MUFA**				
**F2**	C14:1 (Myristoleic)	0.17	0.11	0.08	0.01
	C15:1 (cis-10-Pentadecenoic)	0.31	0.26	0.29	0.03
	C16:1 (Palmitoleic)	32.43	13.69	47.13	3.47
	C18:1n9c (Oleic)	1.84	0.53	2.73	0.22
	C20:1n9 (cis-11-Eicosenoic)	0.09	0.03	0.08	0.02
	Total MUFA	34.85 (29.85%)	14.50	50.31 (32.62%)	3.72
**Low P**	C14:1 (Myristoleic)	0.03	0.00	0.01	0.01
	C15:1 (cis-10-Pentadecenoic)	1.58	0.04	0.45	0.03
	C16:1 (Palmitoleic)	84.97	8.44	20.32	0.10
	C17:1 (cis-10-Heptadecenoic)	0.86	0.22	0.13	0.01
	C18:1n9t (Elaidic)	0.41	0.11	0.34	0.00
	Total MUFA	87.85 (40.65%)	8.37	21.25 (51.09%)	0.12
	**PUFA**				
**F2**	C16:3	19.92	5.54	22.05	1.40
	C16:4	1.23	0.58	0.51	0.06
	C18:2n6c (Linoleic)	1.39	0.52	1.77	0.17
	C18:3n6 (γ-Linolenic)	0.37	0.25	0.67	0.11
	C18:3n3 (α-Linolenic)	0.23	0.06	0.44	0.02
	C18:4	1.42	0.67	2.97	0.50
	C20:4n6 (Arachidonic)	0.54	0.26	0.90	0.17
	C20:5n3 (cis-5,8,11,14,17-Eicosapentaenoic)	17.367	7.87	33.80	6.41
	C22:6n3 (cis-4,7,10,13,16,19-Docosahexaenoic)	3.42	1.28	6.64	0.93

	Total PUFA	45.88 (39.32%)	15.90	69.75 (45.22%)	6.77
**Low P**	C16:2	6.97	2.21	0.88	0.002
	C16:3	7.12	6.48	0.72	0.02
	C16:4	trace	0.00	trace	0.00
	C18:2n6t (Linolelaidic)	2.23	2.98	0.03	0.00
	C18:3n6 (γ-Linolenic)	0.55	0.07	0.34	0.001
	C18:3n3 (α-Linolenic)	0.08	0.01	0.03	0.002
	C18:4	1.67	0.07	0.15	0.002
	C20:3n3 (cis-11,14,17-Eicosatrienoic)	0.04	0.01	trace	0.00
	C20:4n6 (Arachidonic)	trace	0.00	trace	0.00
	C20:5n3 (cis-5,8,11,14,17-Eicosapentaenoic)	19.85	2.43	3.64	0.18
	C22:6n3 (cis-4,7,10,13,16,19-Docosahexaenoic)	5.62	0.98	0.93	0.15
	Total PUFA	44.13 (20.42%)	8.10	6.72 (16.16%)	0.01
